# Regulatory Network Analyses Reveal Genome-Wide Potentiation of LIF Signaling by Glucocorticoids and Define an Innate Cell Defense Response

**DOI:** 10.1371/journal.pgen.1000224

**Published:** 2008-10-17

**Authors:** David Langlais, Catherine Couture, Aurélio Balsalobre, Jacques Drouin

**Affiliations:** Laboratoire de Génétique Moléculaire, Institut de Recherches Cliniques de Montréal (IRCM), Montréal, Quebec, Canada; University of Michigan, United States of America

## Abstract

While the hypothalamo-pituitary-adrenal axis (HPA) activates a general stress response by increasing glucocorticoid (Gc) synthesis, biological stress resulting from infections triggers the inflammatory response through production of cytokines. The pituitary gland integrates some of these signals by responding to the pro-inflammatory cytokines IL6 and LIF and to a negative Gc feedback loop. The present work used whole-genome approaches to define the LIF/STAT3 regulatory network and to delineate cross-talk between this pathway and Gc action. Genome-wide ChIP-chip identified 3,449 STAT3 binding sites, whereas 2,396 genes regulated by LIF and/or Gc were found by expression profiling. Surprisingly, LIF on its own changed expression of only 85 genes but the joint action of LIF and Gc potentiated the expression of more than a thousand genes. Accordingly, activation of both LIF and Gc pathways also potentiated STAT3 and GR recruitment to many STAT3 targets. Our analyses revealed an unexpected gene cluster that requires both stimuli for delayed activation; 83% of the genes in this cluster are involved in different cell defense mechanisms. Thus, stressors that trigger both general stress and inflammatory responses lead to activation of a stereotypic innate cellular defense response.

## Author Summary

Global biological responses involve pleiotropic, general components exhibited by many cells/tissues together with cell-specific responses. Typically, such responses are dependent on multiple signaling pathways that integrate different inputs to trigger concerted tissue/cell responses. In studying LIF action in the context of immune-endocrine regulatory interactions, we found that LIF regulates expression of a surprisingly small number of genes. In contrast, the mapping of LIF-activated STAT3 transcription factor recruitment by genome-wide ChIP-chip led to the identification of a much larger set of putative regulatory sites. In view of the cross-talk between cytokine and glucocorticoid (Gc) signaling in response to stress and inflammation, we investigated the contribution of Gc to LIF action. Interestingly, the discrepancy between the number of LIF-regulated genes and LIF-dependent STAT3 genomic targets was partly explained by widespread Gc potentiation of LIF action. We further show requirement on both signaling pathways to elicit a pleiotropic and stereotypic innate cellular defense response, together with cell-specific responses such as antagonism between cytokines and Gc on expression of pituitary POMC. Thus, this stereotypic innate cell defense response is defined by the convergence of pathways activated by the stress and inflammatory systems.

## Introduction

The pituitary gland is at the center of the hypothalamo-pituitary-adrenal (HPA) axis that mediates the response to stress [1],[2]. Under normal conditions, the stress response is an integrated collection of tissue responses that place the organism in a state of alertness in order to fight or flight in the face of aggression. The output of the HPA axis during the stress response is exerted by circulating glucocorticoids (Gc). Indeed, Gc are synthesized by the adrenals in response to pituitary adrenocorticotropic hormone (ACTH) which itself is responsive to hypothalamic corticotropin-releasing hormone (CRH) that integrates neural inputs into this neuro-endocrine pathway. Gc exert their metabolic effects and a stress response through action on a wide range of tissues including liver, muscle and adipose tissues. The metabolic effects of Gc are profound and failure to maintain Gc levels within the normal range as in Addison disease (hypocortisolism) results in weight loss, muscle weakness, fatigue and low blood pressure. Cushing syndrome is caused by excess Gc and in Cushing disease, this excess is due to pituitary corticotroph adenomas. Cushing syndrome is associated with accumulation of body fat, cardiovascular and metabolic effects that can ultimately lead to hypertension, diabetes and osteoporosis [3]. It is therefore critical that activation of HPA axis and Gc synthesis be restored to normal levels following the stress response. Negative feedback is exerted by Gc themselves both at the level of hypothalamus where they repress transcription of the CRH gene and release of CRH, and at the pituitary level where they repress transcription of the pro-opiomelanocortin (*Pomc*) gene and the release of POMC-derived ACTH [1].

The inflammatory response is a response to biological stresses and various aggressions including those caused by infections [4]. Many effects of the response to inflammation are mediated through cytokines that act on multiple tissues and importantly on the HPA axis. Indeed, inflammation-induced cytokines, such as IL6, stimulate hypothalamic production of CRH and act directly on pituitary corticotroph cells to stimulate *Pomc* gene transcription and ACTH release. LIF, a member of the IL6 family, also contributes to stimulation of POMC expression, both during development and in adult function [5]. At the level of pituitary corticotroph cells, the action of LIF and IL6 are additive with those of hypothalamic CRH [6]. The HPA axis is thus at the center of the so-called immuno-neuroendocrine interface [7].

The action of LIF/IL6 in pituitary corticotroph cells was shown to be mediated in part through activation of STAT3 [8]. STAT3 action on the *Pomc* promoter was mapped to a composite regulatory element that also contains the NurRE, a binding site for dimers of orphan nuclear receptors of the Nur subfamily [9],[10]. The Nur subfamily of orphan nuclear receptors includes NGFI-B (Nur77), NURR1 and NOR1 [11] and it was shown that homodimers or heterodimers between members of this subfamily can activate the NurRE in response to CRH as long as at least one moiety of the dimers is NGFI-B [12]–[14]. Thus, a composite regulatory element integrates LIF/IL6 and CRH signaling.

Gc repress *Pomc* gene transcription and in particular, antagonize *Pomc* activation by CRH and LIF [15]. Feedback repression of the *Pomc* gene by the Gc receptor (GR) is mainly exerted at the level of the NurRE/Stat3 composite regulatory element [16],[17]. GR repression at the NurRE involves a mechanism of trans-repression that depends on protein∶protein interactions between GR and NGFI-B, rather than direct GR contact with DNA [16]. Further, the weak direct interaction between GR and NGFI-B requires the presence of the Swi/Snf ATPase Brg1 for stable formation of a trans-repression complex [18]. Brg1 is also required to recruit HDAC2 to this repressor complex and this repression involves chromatin remodeling. Thus, the NurRE/Stat3 regulatory element of the *Pomc* gene is a critical target for most stimulatory and inhibitory inputs into this system.

In the present work, we have used whole-genome approaches to identify STAT3 target sites as revealed by ChIP-chip analysis using whole-genome tiling arrays [19]–[21] and to correlate these with the transcriptome of LIF and Gc responses. These analyses defined gene clusters that contribute to the repressor effects of Gc on corticotroph cell function, in particular the inhibitory Gc effect on cell proliferation. Most interestingly, the work revealed a class of genes that have delayed responses to LIF+Gc: a large number of these genes contribute to the cell defense response. Using a highly LIF- and Gc-dependent gene of this group, lipocalin 2 (*Lcn2*), we show synergistic recruitment of STAT3 and GR at a genomic regulatory module that integrates LIF and Gc responses. Further, LIF and Gc synergism is exerted on Lcn2 expression and other cell defense genes in various tissues *in vivo* and the gene profile of this action is very similar to that of LPS, a strong inducer of the inflammatory response. Collectively, this work highlights a general cell defense response that is dependent on the combined action of LIF or other cytokines released during inflammatory and immune responses and Gc produced by the HPA axis. This delayed stimulatory Gc action likely overlaps with hepatic acute-phase and innate immune responses [22],[23], and it contrasts with the anti-inflammatory properties of these steroids used therapeutically.

## Results

### LIF/STAT3 Target Genes

**Figure 1:**
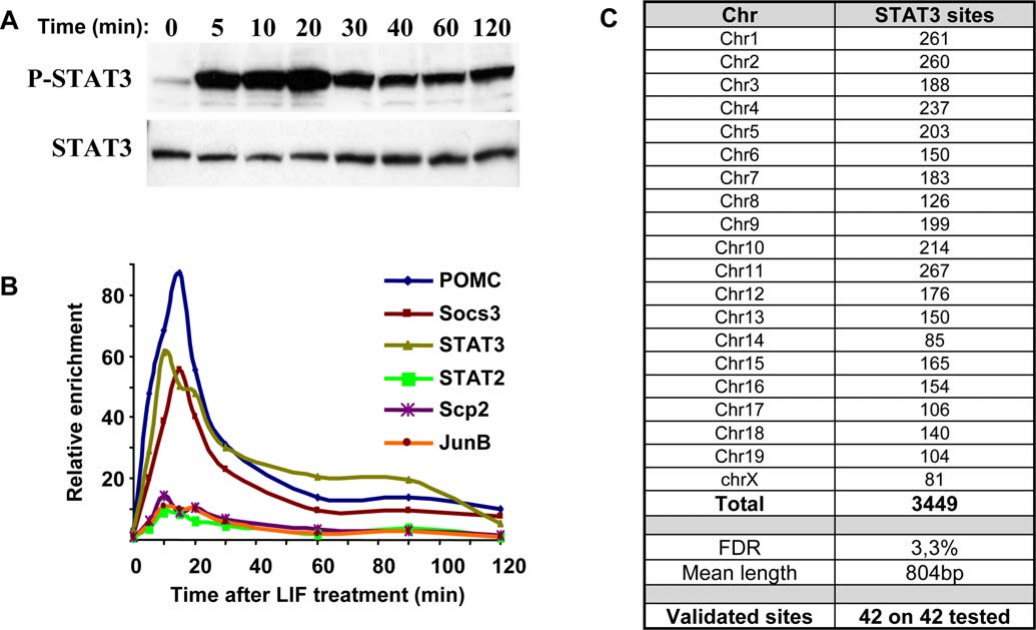
Targets of LIF/STAT3 action. A) The time course of STAT3 activation (phospho- STAT3) was determined in AtT-20 cells following treatment with LIF (10 ng/ml). Western blot analysis of P-STAT3 is compared to total STAT3 protein. B) Time course of STAT3 occupancy on the promoter of known STAT3 target genes determined by ChIP and QPCR. C) Chromosomal distribution of genomic binding sites for STAT3 determined by ChIP-chip analysis of LIF-treated (20 min) AtT-20 cells. Triplicate ChIP samples were analyzed on Affymetrix Mouse Tiling 2.0R Array Sets. Raw data were extracted with GCOS software (Affymetrix) and were analyzed using the MAT software package. STAT3 enrichment peaks were selected on the basis of a *P* value threshold of 10^−5^. Redundant sequence filtering led to the removal of 74 sequences, thus yielding a final count of 3449 STAT3 binding sites. The list of these sites is presented in Table S1. The tiling array results were validated by QPCR analysis of independent ChIPs for 42 loci distributed randomly throughout all chromosomes; all 42 were confirmed. The same loci were used for further studies in Figure 3. FDR, calculated false discovery rate.

In order to assess the cellular response to LIF/STAT3, the time course of STAT3 activation in response to LIF in AtT-20 cells, a model of mouse pituitary corticotroph cells, was determined by Western blot analysis of phospho-STAT3 (Figure 1A). This analysis indicated a peak of phospho-STAT3 at about 20 minutes following LIF treatment. In principle, activated phospho-STAT3 should lead to promoter occupancy of STAT3 target genes and thus the time course of promoter recruitment was assessed by chromatin immunoprecipitation (ChIP) in AtT-20 cells for a panel of STAT3 target genes (Figure 1B). For most of these genes, maximal promoter occupancy was achieved between 10 and 20 minutes after LIF stimulation.

Genomic targets of LIF activated STAT3 were therefore identified by ChIP-chip analysis of AtT-20 cells treated with LIF for 20 minutes. Three independent STAT3 ChIP and control IgG samples were hybridized on the Affymetrix Mouse Tiling 2.0R Array Set, covering the entire non-repetitive mouse genome with a 35 bp resolution. The raw data were processed using the MAT software package [24]. A threshold *P* value of 10^−5^ was used to select peaks of specific STAT3 immunoreactivity throughout the genome, yielding a calculated false discovery rate (FDR) of 3.3% [25]. This analysis revealed a total of 3 449 putative STAT3 target sites in the mouse genome, after removal of 74 sites by redundant sequence filtering (complete list in Table S1). The chromosomal distribution of these sites is shown in Figure 1C. The mean length of genomic regions exhibiting a positive ChIP signal is 804 bp. In order to test the reliability of those results, 42 genomic sites with *P* values ranging from 10^−5^ to 10^−148^ were randomly picked and STAT3 recruitment at each of these sites was tested on separate ChIP using QPCR: all 42 tested sites were confirmed to be positive (Figure 1B–C and data not shown).

### STAT3 Binding Sites Preferentially Localize Close to Transcribed Sequences

**Figure 2:**
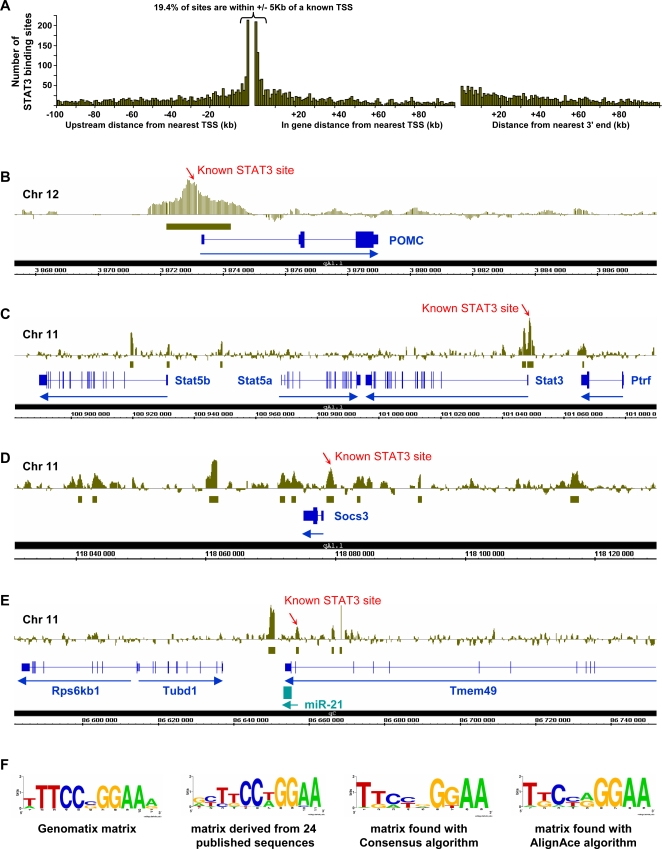
STAT3 genomic binding sites. A) Distances between STAT3 binding peaks determined by whole-genome ChIP-chip and nearest known genes (UCSC mm7 mouse genome assembly). Depending on position relative to the closest gene, data were computed as upstream to nearest TSS (left), relative to the TSS within the body of the gene itself (middle) or relative to the 3′ end of the gene (right). STAT3 binding sites that are outside these three categories were for the most part intergenic and this group constitutes 21.6% of the total number of STAT3 binding sites identified. B) Affymetrix Integrated Genome Browser (IGB) representation of tiling array data for STAT3 recruitment at the *Pomc* locus. In the top diagram, each vertical line represents the MAT score for one 25 bp oligonucleotide probe; each probe is spaced by 10 bp. The green solid horizontal bar indicates the interval of statistically significant (*P*≤10^−5^) STAT3 recruitment. This region contains a documented STAT3 binding site at −387/−379 bp [8],[9]. C) STAT3 binding sites within the *Stat3/Stat5* locus. The upstream region of the *Stat3* gene was previously shown to contain an auto-regulatory STAT3 binding site at position −338/−331 bp [26]. Strong recruitment of STAT3 was observed in this region but also at other positions within the *Stat3/Stat5* locus. Statistically significant peaks (*P*≤10^−5^) of STAT3 binding are marked by the green boxes under the data diagram for tiling microarray data. D) Multiple STAT3 binding sites in the *Socs3* locus including an upstream site that correlates with the previously documented site at −72/−62 bp [27]. E) STAT3 binding sites flanking a microRNA gene, *miR-21*. The STAT3 binding peak at −2801 bp is located near a STAT3 binding site previously identified in human [28]. F) WebLogo representation of known and computed preferred binding site for STAT3. The STAT3 binding site used by the MatBase database (Genomatix) for *in silico* analysis is shown together with a binding site derived from analysis of the 24 published sequences for STAT3 binding. All STAT3 binding regions from the tiling analysis were used to search for redundant DNA motifs, using non-biased algorithms: Consensus and AlignAce. As shown, both algorithms identified similar motifs. No other motif was identified within this dataset.

The position of STAT3 binding sites on the mouse genome was analyzed relative to transcription start sites (TSS) of UCSC known genes. They were mapped either as upstream relative to known TSS, downstream from known TSS within the gene body or relative to the 3′ end of UCSC known genes (Figure 2A). This analysis clearly showed a preferential localization of STAT3 binding sites within 5 kb of TSS, with 19.4% of the total site number within this interval and 9.4% within 1 kb of TSS. Tiling array data for specific loci previously known to have STAT3 binding sites are also shown in Figure 2. For example, the promoter region of the *Pomc* gene is known to have a STAT3 binding site at −387/−379 bp [8]–[10], and the tiling array data show a peak of STAT3 recruitment over this promoter region (Figure 2B). Similarly, the promoter of the *Stat3* gene itself is known to have a STAT3 binding site, and thus is subject to auto-regulation. The tiling array shows a peak of STAT3 recruitment (Figure 2C) that overlaps the reported STAT3 binding site at −338/−331 bp [26]. The *Socs3* gene is involved in negative feedback regulation of STAT3 signaling and the *Socs3* promoter has a STAT3 binding site at −64/−72 bp [27] that overlaps the observed peak of STAT3 recruitment (Figure 2D). In addition to these sites, the tiling array data revealed numerous other STAT3 binding sites in the *Stat3/Stat5* and *Socs3* loci; the biological relevance of these putative regulatory regions will need to be evaluated. Interestingly, STAT3 binding sites were found in close proximity to all Stat genes, except *Stat6*. Finally, STAT3 binding sites were found in the vicinity and promoter region of some microRNA genes, for example around the *miR-21* gene (Figure 2E) that was implicated in the STAT3-dependent growth promotion activity of IL6 [28].

The DNA binding sequence for STAT3 has been defined experimentally through the work of numerous investigators. For example, the binding motif used by the Genomatix software to identify putative STAT3 binding sites is shown in Figure 2F and compared with a consensus that we derived from 24 published genomic STAT3 binding sites. We have used two non-biased algorithms designed to identify recurring motifs within the STAT3-bound DNA fragments (Figure 1C); the AlignAce algorithm and the Consensus algorithm identified a consensus binding motif that is very similar to the previously documented binding sites for STAT3 (Figure 2F). No other motif was found to be enriched within the ensemble of STAT3 genomic targets. We also searched the 3 449 STAT3 target sequences for known transcription factor binding motifs with MatInspector (Genomatix) software and again, we found no other enriched motif compared to 10 randomly picked genomic sequences of the same total length.

### Reciprocal Co-Potentiation of STAT3 and GR Recruitment to Genomic Target Sites

**Figure 3:**
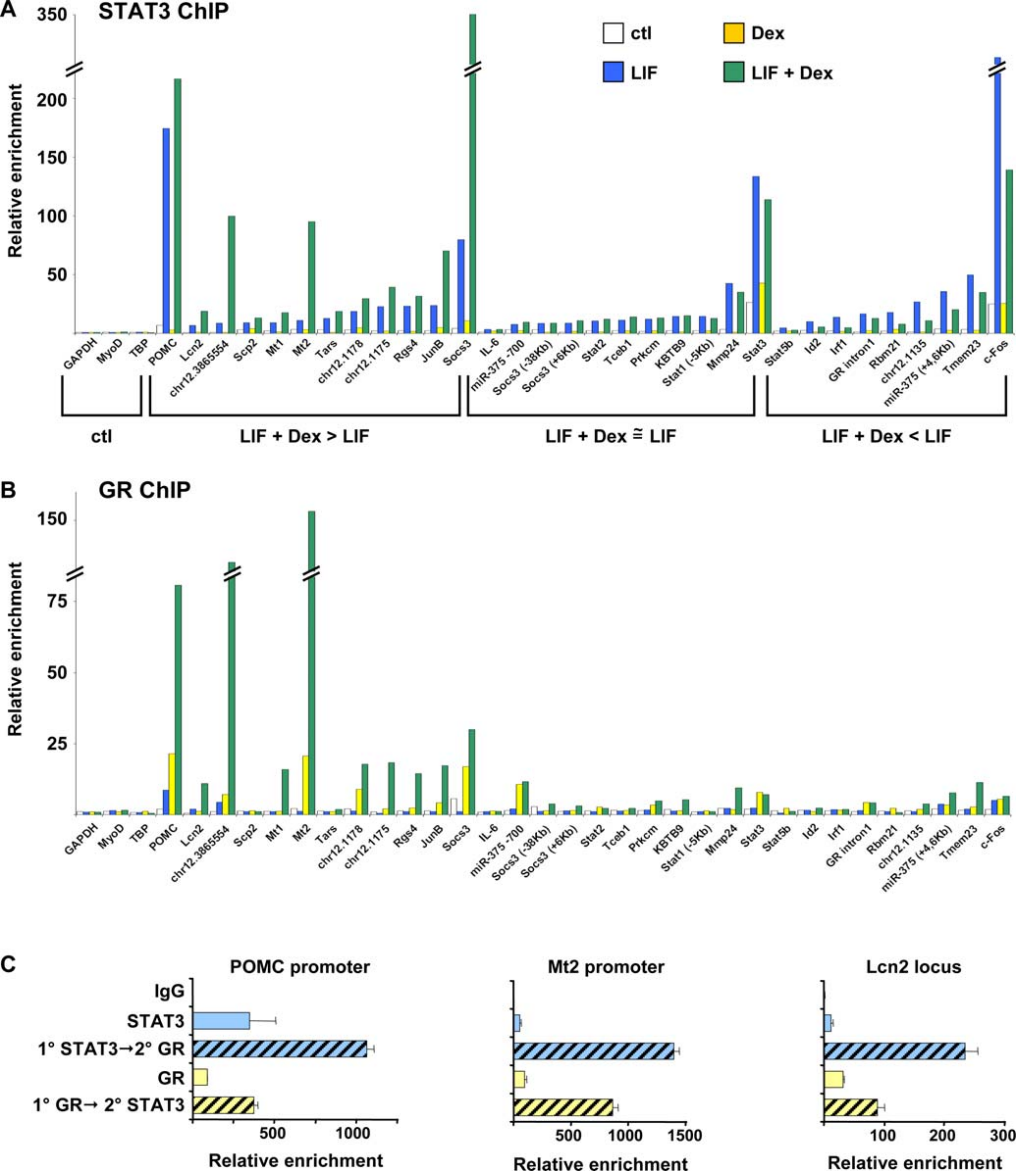
Potentiation of STAT3 and GR recruitment at a subset of LIF/STAT3 target genes. A) A group of 32 genomic STAT3 target sites and 3 control (ctl) loci (*Gapdh*, *Myod* and *Tbp*) were evaluated for STAT3 binding by QPCR analysis of ChIP performed on AtT-20 cells treated for 20 minutes with LIF (10 ng/ml), Dex (10^−7^ M), both or vehicle. Following analysis, genes were re-grouped for presentation in three classes: those for which STAT3 recruitment is greater (≥1.25 fold) in LIF+Dex than LIF-treated cells and those for which this is equal or smaller. B) GR ChIP performed on the same loci as for STAT3. C) Sequential ChIPs were performed for three loci on chromatin isolated from AtT-20 cells treated with LIF and Dex to confirm co-occupancy of STAT3 and GR on the same DNA fragments. Data are shown for single ChIP and for samples immunoprecipitated first with STAT3 and then GR antibody, and the reverse. In each case, data is presented as fold recruitment relative to the IgG sample and normalized by *Gapdh* as QPCR reference.

In AtT-20 cells, the stimulatory effect of LIF on *Pomc* gene transcription is antagonized by Gc and GR. In order to assess whether this antagonism is reflected at the level of STAT3 genomic recruitment, we performed STAT3 ChIP in cells treated either with LIF, the synthetic Gc dexamethasone (Dex) or both for 20 minutes and determined STAT3 recruitment by QPCR for a panel of STAT3 target genes (Figure 3A). While some genes such as *Pomc* showed moderately enhanced STAT3 recruitment in response to LIF+Dex compared to LIF, other genes such as metallothionein 2 (*Mt2*) revealed marked synergism in STAT3 recruitment in cells treated with LIF+Dex (Figure 3A). This suggests that recruitment of one factor potentiates recruitment of the other factor to target regulatory sequences. About a third of tested genes showed greater STAT3 recruitment for LIF+Dex compared to LIF treated cells while another third showed decreased recruitment and the remaining third showed no effect. In order to assess whether potentiation of STAT3 recruitment is reciprocal, similar ChIP analyses were performed for GR recruitment to the same loci and these analyses again showed potentiation of GR recruitment following LIF+Dex treatment for the same subset of genes, such as *Pomc* and *Mt2* (Figure 3B). It is noteworthy that so many randomly chosen STAT3 target loci are also Gc/GR targets. Sequential ChIP were performed for STAT3 and GR on three loci using AtT-20 cells treated with LIF+Dex. These analyses confirmed that for the *Pomc*, *Mt2* and *Lcn2* loci, both GR and STAT3 are present together on the same chromatin fragments (Figure 3C). These data clearly suggest that a subset of LIF target genes may be subject to the combined action of LIF and Gc.

### Synergistic Action of LIF and Glucocorticoids

**Figure 4:**
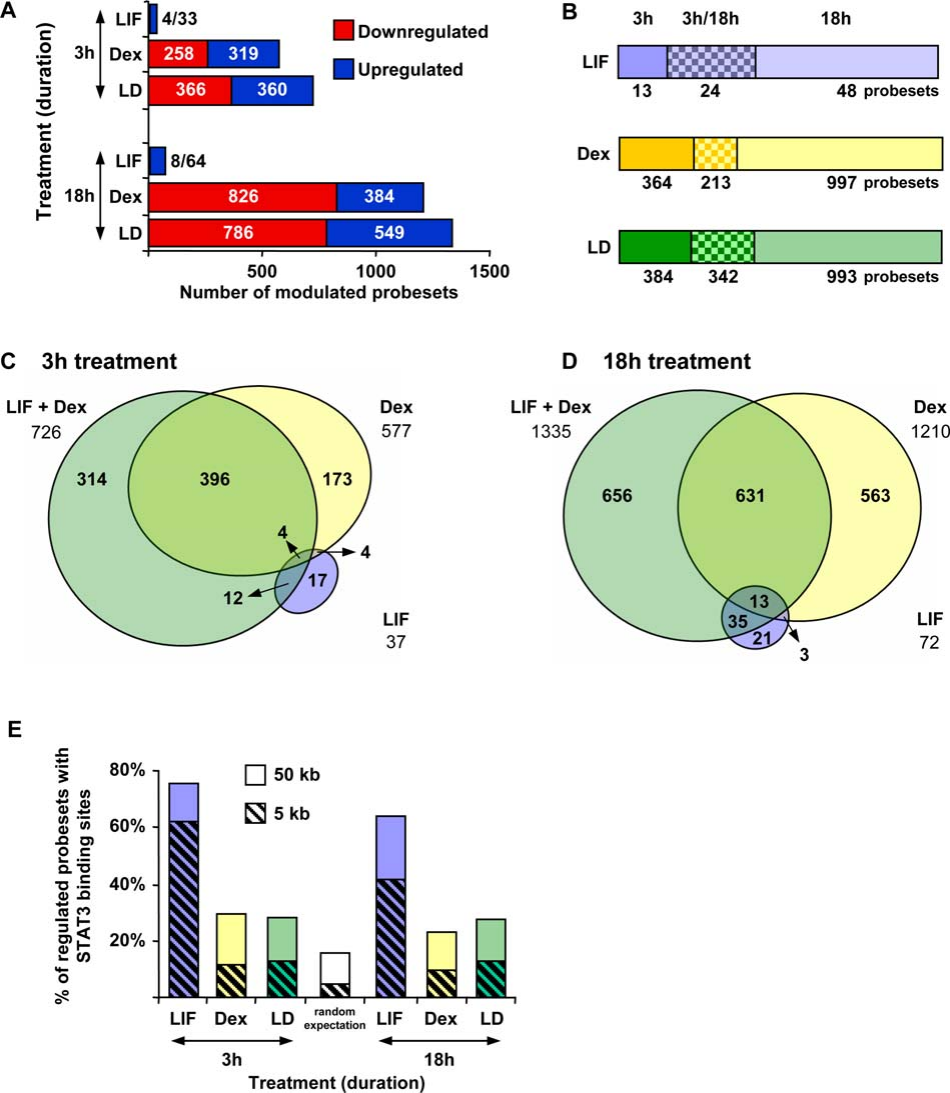
Identification of LIF and glucocorticoid regulated genes. A) AtT-20 cells were treated with LIF, Dex, both or vehicle and total RNA was extracted from cells after 3 h and 18 h of treatment. Affymetrix MOE expression arrays were used to assess expression levels for 45101 probesets in each condition. The expression profiling data were normalized with the GC-RMA algorithm and statistical analysis was measured by Local-pooled-error test (LPE). The replicate variance is <0.001. Changes in gene expression levels in hormone-treated relative to control greater than 2-fold and for *P*≤0.05 were considered statistically significant. A total of 2396 probesets were thus identified, including all treatment conditions (Table S2). The bar histogram represents the number of probesets found to be up or down regulated in each condition. B) Bar diagram representing the number of probesets responding to treatment at either or both time points, for each condition. C–D) Venn diagram showing the overlap of probesets regulated by LIF, Dex or the combination at 3 h and 18 h of treatment. A large number of probesets were found to be uniquely regulated by both agents. E) Percentage of hormone regulated genes (probesets) that have at least one STAT3 binding site in the interval between 5 or 50 kb upstream or downstream of the gene. The random expectation value is calculated on all the genes present on the Affymetrix MOE 2.0 microarray.

In order to correlate STAT3 genomic binding sites with regulation by LIF or Gc of adjacent candidate target genes, we performed expression profiling experiments. Duplicate RNA samples from AtT-20 cells treated with/without LIF and/or Dex for 3 h and 18 h were hybridized on Affymetrix MOE expression arrays. The data were pre-processed using GC-RMA normalization within the FlexArray software [29],[30]. A total of 2 396 regulated probesets were identified (complete data provided in Table S2) following a Local-pooled-error test, using a 2-fold change threshold and a *P* value smaller than 0.05 [31]. The number of genes up or down regulated by these treatments is presented in Figure 4A. Whereas a large number of genes were up and down regulated by Dex, few genes are affected by LIF (mainly up regulated). This low number of modulated genes was unexpected because we identified 3 449 STAT3 binding sites in presence of LIF. Most significantly, a large number of new genes are regulated in response to both LIF+Dex, at both 3 h and 18 h post-treatment (Figure 4C, D). It is noteworthy that early and late response genes are quite different with a limited number of genes showing sustained changes of expression at both 3 h and 18 h (Figure 4B). These data clearly suggest that a class(es) of gene(s) is dependent on both LIF and Gc for regulation.

In order to correlate LIF regulated genes identified in these profiling experiments with genomic sites of STAT3 binding identified by ChIP-chip, we searched for STAT3 binding sites within 5 or 50 kb of the TSS of hormone responsive genes (Figure 4E). This analysis showed that 62/42% of LIF regulated genes have STAT3 binding site within 5 kb of their TSS, and 76/64% within 50 kb of the TSS, at 3 h/18 h respectively. This proportion is smaller for Dex and LIF+Dex-regulated genes, reaching about 30% of genes within 50 kb of TSS. This is higher than the random expectation value of 18%, calculated for all genes on the Affymetrix MOE 2.0 microarray.

### Cell Response to Stress Is Activated by Joint Action of LIF and Gc

**Figure 5:**
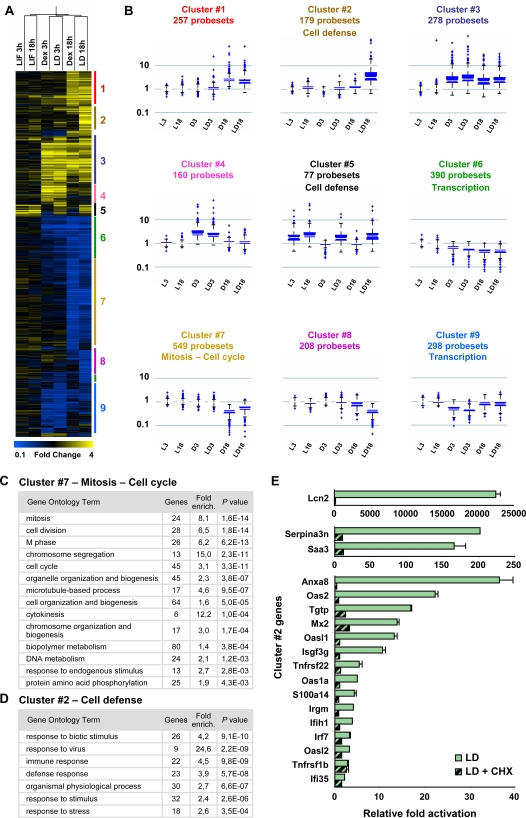
Clustering analysis of LIF and Dex regulated genes. A) Heat map representation of gene clustering identified by global analysis of the expression profiling dataset. The clustering was performed with GeneSpring GX 7.3 using Smooth correlation. B) Box plot representation of Smooth correlation K-means clustering of hormone regulated genes. The analyses required a minimum of 9 clusters in order to represent the different subgroups of genes that were found to be significantly associated. The list of genes in each cluster is presented in Table S3. C) Gene Ontology distribution of genes from cluster #7 that are repressed by Gc at 18 h of treatment, irrespective of the presence of LIF; these genes are highly enriched (*P*≤10^−14^–10^−11^) in cell cycle and mitosis associated functions. The GO gene lists are presented in Table S4. D) Gene Ontology distribution of cluster #2 genes that are upregulated by the joint action of LIF and Dex at 18 h of treatment. These genes are implicated (*P*≤10^−10^–10^−4^) in cell defense response processes (Table S4). Gene Ontology analyses were made using the DAVID web site [32]. E) RT-QPCR analysis of randomly selected cluster #2 genes in AtT-20 cells treated with LIF+Dex in presence (LD+CHX) or absence (LD) of the protein synthesis inhibitor cycloheximide. These genes thus exhibit a secondary protein synthesis-dependent delayed response.

Clustering analysis using Smooth correlation in the Genespring GX 7.3 software was performed on the expression profiling data of hormone-treated AtT-20 cells. A heat map (Figure 5A) of this clustering identified groups of genes that are similarly regulated (Figure 5B). Clustering analysis was performed using the Smooth correlation K means approach. These clusters of co-regulated genes contain from 77 to 549 probesets (Table S3). The DAVID software was used to search for over-represented Gene Ontology (GO) classes of gene functions [32]. Clusters #1, 3, 4 and 8 did not contain significant numbers of genes associated with similar biological processes (GO gene lists in Table S4). Cluster #9 regroups genes that are repressed by Dex at both time points: this cluster contains significant enrichment for genes encoding transcription and nuclear functions (*P*≤10^−5^) and cell processes (*P*≤10^−6^). Interestingly, cluster #7 is highly enriched in genes involved in control of cell cycle and mitosis (*P*≤10^−14^) and these genes (Figure 5C) are primarily repressed by Dex at 18 h (Figure 5A and 5B). It is reassuring to find this cell cycle and mitosis cluster associated with Gc repression since the growth of AtT-20 cells is known to be inhibited by these steroids [33].

The most striking cluster to be identified in this work is represented by the 179 probesets of cluster #2 (Figure 5B). These genes have the particularity of being specifically upregulated at 18 h by the combined action of LIF+Dex, but not by Dex or LIF alone. Gene Ontology analysis of this cluster reveals a highly significant (*P*≤10^−8^) number of genes that are associated with cell defense response (Figure 5D). To a lower extent, we found other genes implicated in cell defense response in cluster #5 (Table S4), which contains the genes activated by LIF at 3 h or 18 h independently of the presence of Gc. The delayed (18 h) response of cluster #2 genes is suggestive of a secondary response. In order to ascertain whether this is the case, we assessed responsiveness to LIF+Dex of a representative panel of cluster #2 genes in the presence/absence of the protein synthesis inhibitor cycloheximide (Figure 5E). This experiment clearly showed that the bulk of this LIF+Dex response is secondary and dependent on *de novo* synthesis of an intermediate regulator(s). Of the genes that are subject to synergistic activation by LIF+Dex, the *Lcn2* gene showed the most striking potentiation.

### *Lipocalin 2*, a Highly LIF- and Glucocorticoid-Dependent Gene

**Figure 6:**
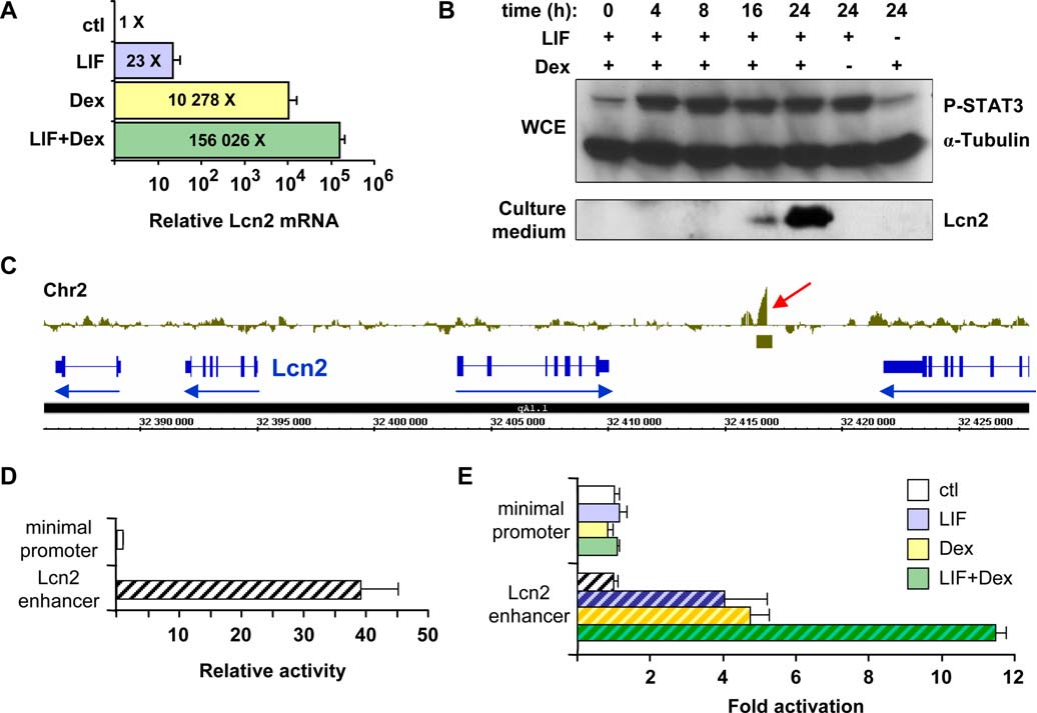
Highly synergistic activation of the lipocalin 2 (*Lcn2*) gene by LIF and glucocorticoids. A) RT-QPCR analysis of *Lcn2* mRNA in AtT-20 cells treated with LIF, Dex or both. Note that the relative mRNA levels are presented on a logarithmic scale. Activation levels relative to control cells are: LIF (23-fold), Dex (10 278-fold) and LIF+Dex (156 026-fold). B) Western blot analysis of Lcn2 induction in AtT-20 cells treated for various times with LIF and Dex as indicated. P-STAT3 levels were visualized by Western blot of whole cell extracts (top panel) whereas secreted Lcn2 was revealed by analysis of culture medium. Lcn2 protein was undetectable in WCE (data not shown). C) Bar representation of STAT3 ChIP-chip data for the *Lcn2* locus from the mouse whole-genome tiling array. The only significant STAT3 recruitment in the vicinity of the *Lcn2* gene was observed in an intergenic region located 22 kb upstream of the *Lcn2* gene (red arrow). D) The STAT3 binding region (1133 bp) of the *Lcn2* locus was cloned upstream of the minimal *Pomc* promoter and assessed for transcriptional activity by transfection into AtT-20 cells. E) The same reporters were assessed for responsiveness to LIF, Dex and LIF+Dex, as indicated. Only *Lcn2* enhancer-containing reporter exhibited hormone responsiveness. Data are presented as means ± s.e.m. of three experiments, each performed in duplicates.

In order to validate the great synergism observed between LIF+Dex effects on *Lcn2* mRNA levels in the microarray analyses, we performed RT-QPCR quantification of *Lcn2* mRNA in AtT-20 cells treated for 18 h with either or both agents. These quantifications indicate that the *Lcn2* gene is responsive to LIF alone (23-fold), highly induced by Dex (10 278-fold), but phenomenally subject to synergism between these two signals (156 026-fold) as shown on a log scale in Figure 6A. This striking upregulation is also revealed by Lcn2 Western blot analysis of AtT-20 cell culture medium (Figure 6B). No STAT3 binding was found at the *Lcn2* promoter (data not shown), but the STAT3 whole-genome ChIP-chip experiment revealed significant enrichment at about 22 kb upstream of the *Lcn2* gene within an intergenic region (Figure 6C) and no other gene is regulated by either LIF and/or Dex in the *Lcn2* vicinity. In order to assess the possibility that this STAT3 binding region might represent a regulatory sequence for *Lcn2* expression, we performed analytical ChIP for STAT3 and GR in this genomic region using cells treated or not with hormones. These data indicated significant potentiation of STAT3 and GR recruitment over this putative regulatory region (Figure 3A, 3B). Sequential ChIP analyses also demonstrate STAT3 and GR co-occupancy on this genomic region (Figure 3C). This −22 kb region may therefore act as a hormone sensitive enhancer for regulation of *Lcn2* expression. In order to test this hypothesis, a luciferase plasmid reporter was constructed with/without the putative 1133 bp enhancer domain and assessed for transcriptional activity upon transfection in AtT-20 cells. This assay revealed marked transcriptional activity of the putative enhancer (Figure 6D) and further, the enhancer-containing reporter plasmid was found to be responsive to LIF, Dex and LIF+Dex treatment (Figure 6E). Thus, these data clearly suggest that an enhancer is present at −22 kb upstream of the *Lcn2* gene and that this enhancer is in part responsible for the marked synergistic activation of *Lcn2* transcription by LIF+Dex. Notwithstanding the likely involvement of a cycloheximide-dependent regulator(s) for long term *Lcn2* induction (Figure 5E), the data implicate direct actions of STAT3 and GR at the *Lcn2* enhancer.

**Figure 7:**
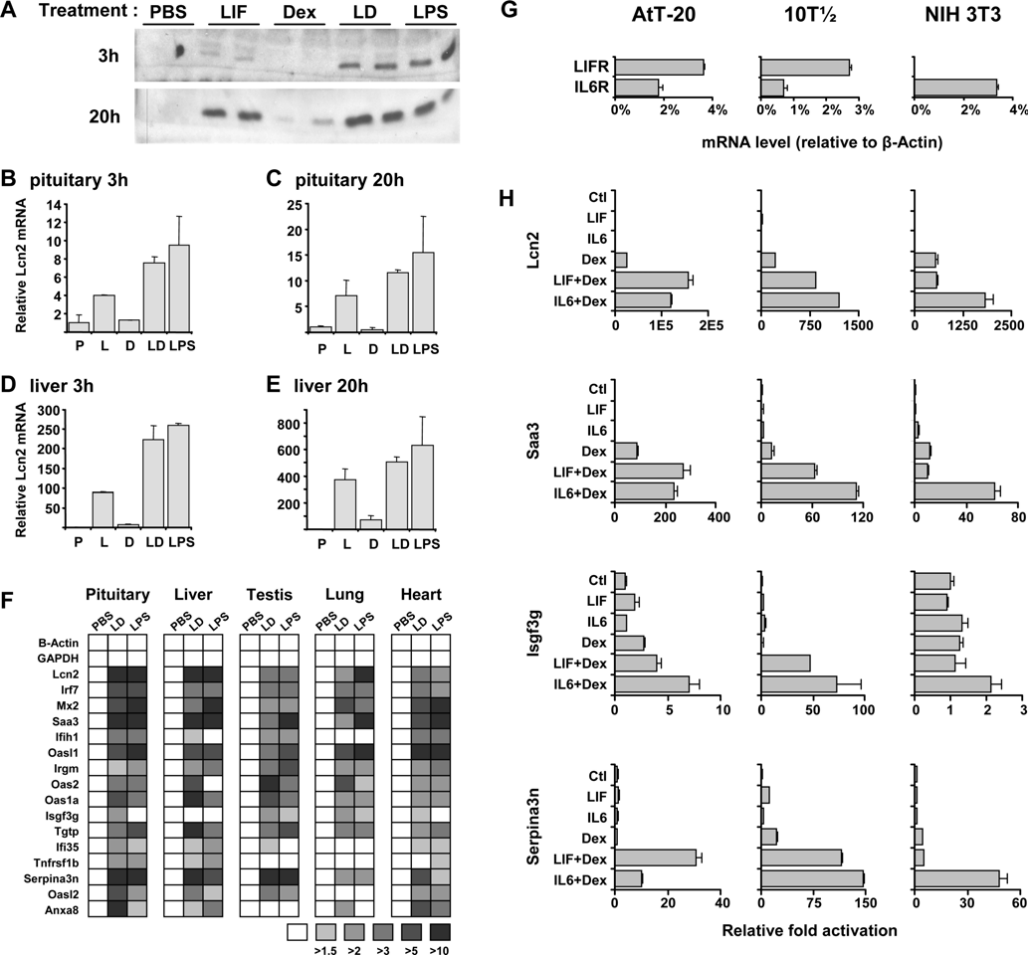
In vivo regulation of LIF and glucocorticoids dependent genes. A) Lcn2 serum levels were measured in mice following injection of LIF (100 µg/kg), Dex (400 µg/kg), or both intraperitoneally at 3 h and 20 h post-treatment. The 20 h group of mice received 5 injections of LIF and/or Dex respectively, in order to maintain hormone plasma levels. A group of mice were also injected with LPS (100 µg/kg) as positive control. Serum Lcn2 revealed by Western blot is shown for 2 mice in each group. B) Pituitary *Lcn2* mRNA levels were measured by RT-QPCR at 3 h post-treatment in mice treated with vehicle (C), LIF (L), Dex (D), LIF+Dex (LD) or LPS, as indicated. C) Pituitary *Lcn2* mRNA levels at 20 h post-treatment. Note different scale relative to B. D) Liver *Lcn2* mRNA levels assessed by RT-QPCR at 3 h post-treatment. E) Liver *Lcn2* mRNA at 20 h post-treatment. F) Genes from cluster #2 implicated in cell defense mechanisms (GO analysis) were randomly picked and the relative abundance of their mRNA was assessed by RT-QPCR in pituitary, liver, testis, lung and heart of mice 20 h after injection of vehicle (PBS), LIF+Dex (LD) or LPS. G) LIF and IL6 receptor mRNA levels relative to β-Actin mRNA as measured by RT-QPCR in untreated AtT-20, 10T½ and NIH 3T3 cells. H) Synergistic activation of *Lcn2* and three cluster #2 genes by LIF+Dex and IL6+Dex treatment in AtT-20, 10T½ and NIH 3T3 cells.

Lcn2 is a secreted protein that is present in blood and its plasma concentration is greatly enhanced following bacterial challenges [34],[35]. In order to test whether LIF+Dex also stimulate *Lcn2* expression *in vivo*, mice were injected with either LIF, Dex or LIF+Dex and analyzed for serum Lcn2. The effect of LIF+Dex was compared to the documented stimulation of Lcn2 expression by lipopolysaccharides O127:B8 (LPS). While Dex on its own did not stimulate serum Lcn2 at 3 h of treatment, injection of LIF led to a small increase in serum Lcn2 but the combined LIF+Dex treatment was even more effective, approaching the response obtained with LPS injection (Figure 7A). At 20 h of treatment, a small response to Dex was observed but again the greatest increase was observed in LIF+Dex treated mice.

Circulating Lcn2 is likely produced by a variety of sources including liver [34]. It is therefore possible that the synergistic stimulation of *Lcn2* gene expression observed in AtT-20 cells may be a reflection of a general cellular response to these agents. In order to test this, RT-QPCR was used to measure *Lcn2* mRNA levels in pituitary and liver of mice injected with LIF, Dex and LIF+Dex (3 and 20 h), together with a reference group of mice injected with PBS or LPS (Figure 7B to 7E). These data indicate that the synergistic action of LIF+Dex is not unique to the pituitary. Liver production of Lcn2 could thus account for a significant proportion of blood Lcn2 observed in animals treated with LIF+Dex.

To assess whether the cell defense mechanism activated in AtT-20 by LIF+Dex (cluster #2) is active and generalized *in vivo*, we randomly selected genes within this cluster. mRNA levels were measured by RT-QPCR in five tissues (pituitary, liver, testis, lung and heart) from mice treated for 20 h with PBS, LIF+Dex or LPS. As above, this experiment was performed in mice that have normal Gc levels using a pharmacological dose of Dex together with LIF. In all five tissues, the two treatments produced comparable patterns of gene activation (Figure 7F). It thus appears that the cell defense mechanisms activated by LIF+Dex are very similar to those activated by LPS, in agreement with the stimulatory effect of LPS on cytokines, ACTH and Gc [36]. Many genes synergistically activated by LIF+Dex are part of the hepatic acute-phase and innate immune response [22],[23].

In view of this widespread *in vivo* response, we verified whether similar responses would be observed in cell lines other than AtT-20. Furthermore, we tested the responses to the LIF-related cytokine IL6 that is also induced during the inflammatory response. We used the 10T½ cells that co-express the LIF and IL6 receptors, like AtT-20 cells, but also the NIH 3T3 cells that only express the IL6 receptor, as shown by RT-QPCR (Figure 7G). These analyses showed LIF+Dex as well as IL6+Dex synergism in all three cell lines (Figure 7H).

## Discussion

The present work was undertaken to define the action of LIF and related cytokines such as IL6 on corticotroph function in the context of immuno-neuroendocrine interactions. Surprisingly, LIF signaling on its own was found to modulate a very limited gene subset. Indeed, most LIF-regulated genes are similarly activated at 3 h and 18 h, they are found in cluster #5 (77 probesets representing 57 unique genes) and many have been involved in corticotroph signaling and *Pomc* transcription, such as *Jak3*, *Stat1*, *Stat3*, *Socs3*, *Junb*, *c-Fos*, *Cebpδ* (Table S3). The majority of these genes recruit STAT3 close to their TSS (Figure 4E) and they contain a canonical STAT3 binding site (Figure 2F). Collectively, they define a pathway for LIF/STAT3-dependent activation of transcription but the small number of genes involved contrasts with the much larger number of genomic STAT3 recruitment sites (Figure 1C). This discrepancy is largely explained by the great number of LIF-sensitive genes that are potentiated by Gc (Figure 4). In contrast to LIF, Gc on their own affect a large number of genes, consistent with other genomic studies of Gc action [37],[38]. Many of these genes respond transiently to Dex either at 3 h (cluster #4 genes are activated, cluster #9 repressed) or at 18 h (cluster #1 genes are activated and #7 repressed) but other gene clusters exhibit sustained changes in expression (activation for cluster #3 and repression for cluster #6). But the most interesting gene clusters to arise from this analysis show delayed (18 h) responses that require both Dex and LIF (cluster #2 for activated genes, cluster #8 for repressed genes). In particular, cluster #2 is enriched in genes involved in different cellular responses to aggression or stress, including genes that are part of the innate immune response and of the hepatic acute-phase response.

### Mechanism of STAT3 Action

The mapping of STAT3 binding sites on the mouse genome in LIF-stimulated cells identified 3 449 high confidence sites (Figure 1). This number stands in stark contrast with the relatively limited number of LIF-regulated mRNAs identified in profiling experiments (Figure 4). Although it is possible that a large number of target genes are regulated less than the 2-fold threshold of expression profiling data, it is more likely that this small number of LIF-regulated genes reflects the dependence of STAT3 on other transcription factors for activity. This action includes a moderate stimulatory effect on *Pomc* gene expression: within the context of *Pomc* regulation, LIF action is mostly meaningful in association with the stimulatory action of CRH signaling and the downstream Nur orphan nuclear receptors [10].

Nonetheless, it appears that activation of STAT3 by phosphorylation (Figure 1A) leads to promoter occupancy of a large number of target genes (Figure 1B, 1C), independently of other signaling pathways. These STAT3 targets include cell-specific genes such as *Pomc* (Figure 2B) and genes involved in STAT3 signaling itself (Figure 2C, 2D). The STAT3 target genes defined through ChIP-chip analysis also include a large number of genes that are co-regulated by Gc. Independently of this co-regulation, non-biased analysis of STAT3 genomic binding regions only revealed one conserved sequence motif, the STAT3 binding site itself (Figure 2F). This conserved motif is entirely consistent with the previously defined STAT3 binding site [27],[39]. It is noteworthy that this analysis did not reveal enrichment of any other motif: it might have been expected that some transcription factor binding motifs might have been enriched in association with STAT3 targets since STAT3 has already been shown to act in association with a variety of factors including GR [40]. Failure to detect particular enrichment of one binding motif with STAT3 binding sites may reflect the fact that STAT3 binding sites is associated with a large array of conserved binding motifs for many structural classes of DNA binding proteins and/or that these other factors act by protein∶protein interactions with STAT3. The localization of binding peaks within STAT3 binding regions corresponded quite closely to the position of known STAT3 binding sites (Figure 2B to 2E). For example in the *Pomc* promoter (Figure 2B), a binding peak was observed at −465 bp whereas the published STAT3 binding site is located at −387/−379 bp [8]–[10].

### Potentiation of LIF Action by Gc

A surprising finding of this study has been the large number of genes that exhibit potentiation of LIF effects (activation or repression) by Gc (cluster #1, 2, 7 and 8). The Venn diagrams (Figure 4C and 4D) clearly illustrate the large number of genes that are subject to Gc potentiation of LIF activity. Interestingly, a similar proportion (about 2/3) of randomly chosen STAT3-binding loci showed enhancement or antagonism of STAT3 recruitment in presence of LIF+Dex compared to LIF alone (Figure 3A). Also, many of these loci showed enhanced GR recruitment in LIF+Dex compared to Dex-treated cells (Figure 3B). The potentiation of GR recruitment to STAT3 loci may involve direct protein interactions between these effectors as such interactions have been documented [41]. Direct STAT3:GR interactions may cause transcriptional synergism [41] but they may also reflect transcriptional antagonism as observed for trans-repression of LIF and/or CRH-induced *Pomc* transcription by GR. Indeed, Gc repress *Pomc* transcription without direct DNA binding by GR: the present work showed enhanced GR and STAT3 recruitment to the *Pomc* promoter in Dex+LIF-treated cells compared to Dex or LIF alone (Figure 3A, B) and we have similarly showed enhanced NGFI-B and GR recruitment to this promoter in CRH+Dex-treated cells compared to either treatment [18]. The potentiation of genomic recruitment of one factor by another is thus a clear indication of transcriptional interactions, but it does not predict whether an interaction may be synergistic or antagonistic on transcription.

### Inhibition of Cell Cycle and Mitosis by Glucocorticoids

In addition to its repressor effect on *Pomc* transcription [42], Gc inhibit the growth of AtT-20 cells [33]. Cluster #7 genes are repressed by Dex at 18 h but not 3 h irrespective of the presence of LIF and it is enriched in genes involved in cell cycle control and mitosis (Figure 5A–C). This gene cluster therefore contains the ensemble of gene functions that may work coordinately to repress cell proliferation. It will be interesting to assess whether a similar group of genes is also involved in the growth inhibitory effects of Gc on immune or other cells.

### The Cell Defense Response

A unique cluster of genes was identified in the present work and is represented by cluster #2 (Figure 5D). This 179 probesets (150 genes) cluster is highly enriched in genes involved in cell defense response. Upon removal of 40 genes of unknown function, the remaining 110 genes with known or suspected function were queried for involvement in various processes. Of these, a total of 91 genes were previously associated with various cell defense mechanisms, such as innate responses to viruses or to bacteria, or acute phase response. This group thus represents 83% of genes with documented function in cluster #2. The group includes genes of the innate response to viral infection that are interferon induced (ISGs) [43]: examples of this group include the six 2′-5′-oligoadenylate synthetase (*Oas*) genes, the *Mx1* and *Mx2* genes, *Irf7* and *Pkr* (Figure S1). Interestingly, the interferon genes themselves and Toll-like receptors were not induced by LIF+Dex. Similarly, the bacterial infection and acute phase response genes [44],[45] *Tpl2*, *Saa3*, Haptoglobin and Serpina3 were all found in cluster #2 but the α2-macroglobulin gene was not. It should be mentioned however that other ISGs and cell defense genes were induced in these experiments under different regulatory modalities and therefore they are found in clusters other than #2. The genes of cluster #2 thus represent an innate defense mechanism that is triggered by joint activation of the inflammatory response and HPA axis. This innate cell defense response may be evolutionary conserved as it has been suggested for the functions of *Mx* and *Oas* genes [46],[47].

The most striking example of a LIF+Dex-dependent gene is *Lcn2* that is induced more than 150 000-fold in AtT-20 cells (Figure 6A). Whereas the *Lcn2* promoter does not exhibit any STAT3 or GR recruitment (Figure 6C and data not shown), their activities are likely conferred, at least in part, upon the *Lcn2* gene by a putative enhancer element identified 22 kb upstream of the *Lcn2* gene (Figure 6C–E). Interestingly, the putative *Lcn2* enhancer exhibits potentiation of GR binding upon LIF/STAT3 action and the reverse (Figure 3). However, it is clear that direct action of STAT3 and GR on the *Lcn2* locus is not the only mechanism of activation since at 18 h post-stimulation, most of the response to LIF+Dex is dependent on *de novo* protein synthesis (Figure 5E). In fact, most of cluster #2 genes exhibit an analogous secondary response.

*Lcn2* regulation thus exemplifies a cell defense response that appears to be shared by many cells and tissues [48],[49]. We have ascertained this *in vivo* by injection of LIF, Dex, or both in normal mice and compared these responses with LPS challenge in pituitary and liver. Lcn2 expression was induced by LIF in both tissues and Dex treatment exerted synergistic activation at 3 h post-treatment (Figure 7A–E). Less synergism of Dex action with LIF was observed *in vivo* compared to tissue culture cells (Figure 6A), but the *in vivo* experiments were conducted in mice with normal adrenal function and Gc levels.

In order to test the responsiveness of cluster #2 genes in various tissues *in vivo*, a similar experiment was conducted in mice injected with LIF+Dex compared to LPS-injected animals. As shown graphically in Figure 7F, the response patterns to these agents are similar in five tissues. It is noteworthy that tissues not usually associated with the acute phase response, share this response pattern. These conclusions are also supported by experiments using different cell lines (Figure 7G and 7H). Thus, LIF/IL6 and Gc appear to elicit an innate cell defense response. With regards to Gc, this positive action has been interpreted as pro-inflammatory [22] but it may be more appropriately interpreted as a local cell defense response that is distinct and complementary to the systemic anti-inflammatory actions of Gc. It is interesting to suggest that the innate cell defense response identified in the present work may constitute an ancestral defense mechanism.

## Materials and Methods

### Cell Culture and Transfection

AtT-20 cells were maintained in DMEM supplemented with 10% fetal bovine serum and antibiotics. The cells were transfected with 500 ng of luciferase reporter construct using Lipofectamine reagent (Invitrogen). The following day, cells were stimulated for 4 h with either PBS as vehicle, LIF 10 ng/ml (Chemicon), dexamethasone (Dex) 10^−7^ M (Sigma), or a combination of LIF+Dex.

### Western Blots

Whole cell extracts (WCE) were prepared and analyzed on SDS-PAGE as described [18]. Western blots were revealed using STAT3 (sc-482), phospho-STAT3 (sc-7993), α-Tubulin (sc-32293) and Lcn2 (sc-50351) antibodies from Santa Cruz Biotechnology.

### Chromatin Immunoprecipitation (ChIP), Sequential ChIP and QPCR

AtT-20 cells were grown to 60–70% confluence and stimulated with 10 ng/ml LIF and/or 10^−7^ M Dex for 20 min. ChIP were performed as described previously [50], with little modifications. Briefly, chromatin was crosslinked with 1% formaldehyde added directly to the culture medium (5 min at room temperature). Crosslinking was stopped with glycine 125 mM in PBS for 5 min, followed by chromatin preparation. Sonicated chromatin was immunoprecipitated with either rabbit IgG (Sigma G2018), GR (sc-1004) or a combination of phospho-STAT3 (sc-7993) and STAT3 (sc-482) antibodies and collected using protein-A/G beads (Santa Cruz Biotechnologies). After washes and decrosslinking, DNA was purified using QIAquick columns following manufacturer's directives. For sequential ChIP, chromatin immunoprecipitates were gently eluted with elution buffer (10 mM Tris-HCl pH8, 1% SDS) for 20 min at 65°C. Supernatants were diluted to 0.5% SDS, 0.5% Triton, 0.05% NaDOC, 10 mM Tris-HCl pH8 and 140 mM NaCl, and complemented with 0.5 mg/ml BSA, 0.05 mg/ml yeast tRNA and 0.025 mg/ml phage λ DNA. The second immunoprecipitation was performed as described above for single ChIP. Enrichment was assed by QPCR with Qiagen QuantiTect SYBR green PCR kit. The list of oligonucleotides used is available upon request.

### Whole-Genome Tiling Arrays

Three independent STAT3 and control IgG ChIP samples were amplified, fragmented, biotin labeled and hybridized on Affymetrix Mouse Tiling 2.0R Array Set as recommended by the company. Raw data were processed with the MAT software [24] to calculate peak intensity and determine statistically significant enrichment of specific genomic regions. A *P* value cut-off of 10^−5^ was applied and redundant sequences were subtracted following BLAT search. Thus, the STAT3 whole-genome ChIP-chip yielded 3 449 sites with a predicted false discovery rate (FDR) of 3.3%.

### Binding Motif Analyses

*De novo* motif analyses were done using two different sequence alignment algorithms. First, 800 bp masked sequences were retrieved from UCSC genome browser for each of the STAT3 binding sites: those included 400 bp upstream and downstream of MAT defined enrichment peaks. These sequences were processed using AlignAce [51] and Consensus [52]. The graphical representation of the position weight matrices obtained from these analyses were generated with WebLogo [53].

The same sequence set was challenged against all known transcription factor binding motifs using the MatInspector software (Genomatix). The resulting occurrence of each motif was compared to the mean number of predicted binding sites in 10 randomly picked genomic sequence sets.

### RNA and Expression Arrays

Total RNA was extracted from AtT-20 cells previously treated for 3 or 18 h with vehicle, 10 ng/ml LIF and/or 10^−7^ M Dex, using RNeasy columns (Qiagen). Two biological replicates of each condition were hybridized on Affymetrix MOE 430 2.0 arrays, except for Dex 18 h that was hybridized on the previous version of MOE A and B arrays. Hybridization and scanning were done at the McGill University and Genome Québec Innovation Centre. Data were normalized using GC-RMA [29],[30] on the FlexArray application. The variance between replicates is smaller than 0.001. We used the Local-pooled-error test (LPE) to assess differential gene expression between control and hormone treated cells [31]. Gene expression with fold changes greater than 2 (*P*≤0.05) were considered significant.

Genes from cluster #2 were picked randomly for RT-QPCR validation. AtT-20 cells were treated with LIF+Dex (10 ng/ml and 10^−7^ M respectively) in presence or absence of cycloheximide at 10 µg/ml (Sigma). We also treated AtT-20, 10T½ and NIH 3T3 cells with LIF (10 ng/ml), IL6 (10 ng/ml), Dex 10^−7^ M alone or in combination for 18 h. Total RNA was extracted as described above and gene expression was quantified with the Qiagen OneStep RT-QPCR kit.

### Clustering and Gene Ontology Analysis

The genes with expression changes in at least one condition (LIF, Dex, LIF+Dex, at 3 h or 18 h) were uploaded into GeneSpring GX 7.3 software (Agilent) for analysis. Smooth correlation was used to do unbiased clustering. Following this, K-mean clustering using Smooth correlation was used to separate genes with the same expression reactivity. We determined that 9 clusters is the most segregating setting for our dataset. The gene lists extracted from those 9 clusters were uploaded into the DAVID website [32] to search for enriched biological processes. The Affymetrix MOE 430 2.0 gene list was used as reference. Thresholds were set at a minimum of 5 genes per Gene Ontology class and a *P* value ≤ 0.001.

### In Vivo Experiments

Groups of six CD1 male mice aged between 10 and 14 weeks were injected intraperitoneally with either PBS, 100 µg/kg LIF, 400 µg/kg Dex, LIF+Dex or 100 µg/kg LPS (O127:B8, Sigma) and sacrificed after 3 h. Similar groups were sacrificed at 20 h following 5 injections, except for LPS (only one LPS injection and 4 PBS injections). Mice were anaesthetized with 0.025 ml/g of avertin 2.5%. 1 ml of blood was collected by cardiac puncture. Serum proteins (100 µg) were loaded onto SDS-PAGE and Lcn2 protein was revealed by Western blot. Lcn2 is a small 26 kDa protein and the upper part of gels was stained with Coomassie blue as loading control. Pituitary, liver, testis, lung and heart were dissected out following sacrifice. Total RNA was extracted from these tissues using RNeasy column as described by Qiagen. cDNA was produced using SuperScript III (Invitrogen) and gene expression was measured by QPCR with Qiagen QuantiTect SYBR green. *Lcn2* and other mRNA levels were normalized in respect to β-actin mRNA. The oligos sequences are available upon request. Animal experimentation was approved by the IRCM Animal Care and Use Committee, in conformity with regulations of the Canadian Council on Animal Care.

## Supporting Information

Figure S1 List of 91 genes from cluster #2 with known/suspected cell defense function.
(0.07 MB PDF)

Table S1 List of genomic STAT3 binding regions selected for a *P* value threshold of 10^−5^ after analysis with MAT algorithm (mouse mm7 assembly).
(0.47 MB XLS)

Table S2 List of LIF and/or glucocorticoid regulated genes.
(1.01 MB XLS)

Table S3 List of gene annotations attributed by DAVID web site for each cluster.
(1.06 MB XLS)

Table S4 Gene Ontology analyses of the nine gene clusters.
(0.13 MB XLS)
