# Supplementary material for: Regulatory Network Analyses Reveal Genome-Wide Potentiation of LIF Signaling by Glucocorticoids and Define an Innate Cell Defense Response
Source: PLoS Genet. 2008 Oct 17;4(10):e1000224. doi: 10.1371/journal.pgen.1000224 (PMC2562516; doi:10.1371/journal.pgen.1000224)
Supplement: Figure S1 — List of 91 genes from cluster #2 with known/suspected cell defense function. (0.07 MB PDF) [file pgen.1000224.s001.pdf]

Figure S1

List of 91 genes from cluster #2 with known/suspected cell defense function

GeneID	Affy probeset	GeneName	GeneSymbol
NM_145211	1424775_at	2'-5' oligoadenylate synthetase 1a	Oas1a
NM_001083925	1425119_at	2'-5' oligoadenylate synthetase 1b	Oas1b
NM_145227	1425065_at	2'-5' oligoadenylate synthetase 2	Oas2
NM_145226	1425374_at	2'-5' oligoadenylate synthetase 3	Oas3
NM_145209	1424339_at	2'-5' oligoadenylate synthetase-like 1	Oasl1
NM_011854	1453196_a_at	2'-5' oligoadenylate synthetase-like 2	Oasl2
NM_007403	1416871_at	a disintegrin and metallopeptidase domain 8	Adam8
NM_008032	1443963_at	af4/fmr2 family, member 2	Aff2
NM_007447	1438936_s_at	angiogenin, ribonuclease a family, member 1	Ang
NM_013473	1417732_at, 1425789_s_at	annexin a8	Anxa8
NM_009711	1432032_a_at	artemin	Artn
NM_009735	1452428_a_at	beta-2 microglobulin	B2m
NM_183162	1435454_a_at	cdna sequence bc006779	BC006779
NM_153576	1451610_at	cdna sequence bc024561	Vcc1
NM_001033419	1441120_at	cea-related cell adhesion molecule 16	Ceacam16
NM_170684	1433715_at	copine vii	Cpne7
NM_172689	1436562_at	dead (asp-glu-ala-asp) box polypeptide 58	Dxd58
NM_010050	1418938_at, 1426081_a_at	deiodinase, iodothyronine, type ii	Dio2
NM_001013371	1435208_at, 1439825_at	deltex 3-like (drosophila)	Dtx3l
NM_194067	1454757_s_at	dna segment, chr 12, erato doi 647, expressed	ISG12a
NM_011163	1422005_at, 1422006_at	eukaryotic translation initiation factor 2-alpha kinase 2	Eif2ak2 (PKR)
NM_134151	1433775_at	expressed sequence c77080	Yars
NM_017370	1448881_at	haptoglobin	Hp
NM_008326	1418825_at	immunity-related gtpase family, m	Irgm
NM_008394	1421322_a_at	interferon dependent positive acting transcription factor 3 gamma	Irf9
NM_018738	1417141_at	interferon gamma induced gtpase	Igtp
NM_026820	1424254_at	interferon induced transmembrane protein 1	Ifitm1
NM_025378	1423754_at	interferon induced transmembrane protein 3	Ifitm3
NM_027835	1426276_at	interferon induced with helicase c domain 1	Ifih1
NM_019440	1417793_at	interferon inducible gtpase 2	Iigp2
NM_016850	1417244_a_at	interferon regulatory factor 7	Irf7
XM_001471685	1431591_s_at	interferon, alpha-inducible protein	Isg15
NM_027320	1424617_at, 1459151_x_at	interferon-induced protein 35	Ifi35
NM_172161	1436507_at	interleukin-1 receptor-associated kinase 2	Irak2
NM_013571	1419823_s_at	kinase suppressor of ras 1	Ksr1
NM_023125	1416676_at, 1426045_at	kininogen 1	Kng1
NM_010708	1421217_a_at	lectin, galactose binding, soluble 9	Lgals9
NM_011175	1448883_at	legumain	Lgmn
NM_178665	1436714_at	lim domain containing preferred translocation partner in lipoma	Lpp
NM_023463	1422749_at	lymphocyte antigen 6 complex, locus g6c	Ly6g6c
NM_010693	1425396_a_at	lymphocyte protein tyrosine kinase	Lck
NM_080638	1448618_at, 1456586_x_at	major vault protein	Mvp
NM_007746	1419208_at	mitogen activated protein kinase kinase kinase 8	Map3k8
NM_139300	1425506_at	myosin, light polypeptide kinase	Mylk
NM_010846	1451905_a_at	myxovirus (influenza virus) resistance 1	Mx1
NM_013606	1419676_at	myxovirus (influenza virus) resistance 2	Mx2
NM_026012	1429127_at	neurotrophin receptor associated death domain	Nradd
NM_019401	1425719_a_at	n-mcy (and stat) interactor	Nmi
NM_133249	1449945_at	peroxisome proliferative activated receptor, gamma, coactivator 1 beta	Ppargc1b
NM_008876	1457252_x_at	phospholipase d2	Pld2
NM_008872	1415806_at	plasminogen activator, tissue	Plat
NM_011113	1452521_a_at	plasminogen activator, urokinase receptor	Plaur
XM_001476537	1452178_at	plectin 1	Parp10
NM_027514	1423903_at, 1423904_a_at, 1451160_s_at, 1423905_at	poliovirus receptor	Pvr
NM_145619	1426210_x_at, 1445888_x_at	poly (adp-ribose) polymerase family, member 3	Parp3

NM_030253	1416897_at	poly (adp-ribose) polymerase family, member 9	Parp9
NM_010610	1425987_a_at	potassium large conductance calcium-activated channel, subfamily m, alpha member 1	Kcnma1
NM_021384	1421008_at, 1421009_at, 1436058_at	radical s-adenosyl methionine domain containing 2	Rsad2
NM_023386	1418580_at	receptor transporter protein 4	Rtp4
NM_029821	1431786_s_at, 1455186_a_at	riken cdna 1190003j15 gene	1190003J15Rik
NM_001039530	1451564_at	riken cdna 1600029o10 gene	Parp14
NM_001081156	1453008_at	riken cdna 2300002d11 gene	2300002D11Rik
XM_911308	1424518_at	riken cdna 2310016f22 gene	Apol9a
NM_023680	1422038_a_at, 1426095_a_at, 1442590_at	riken cdna 2810028k06 gene	Tnfrsf22
NM_030150	1420768_a_at, 1451426_at	riken cdna b430001i08 gene	Dhx58
NM_025393	1449166_at	s100 calcium binding protein a14	S100a14
NM_026416	1425560_a_at	s100 calcium binding protein a16	S100a16
NM_011311	1424542_at	s100 calcium binding protein a4	S100a4
NM_011414	1448377_at	secretory leukocyte peptidase inhibitor	Slpi
NM_009252	1419100_at	serine (or cysteine) peptidase inhibitor, clade a, member 3n	Serpina3n
NM_011315	1450826_a_at	serum amyloid a 3	Saa3
NM_009283	1450033_a_at, 1450034_at	signal transducer and activator of transcription 1	Stat1
NM_133221	1417811_at	solute carrier family 24 (sodium/potassium/calcium exchanger), member 6	Slc24a6
NM_009579	1422786_at, 1436164_at	solute carrier family 30 (zinc transporter), member 1	Slc30a1
NM_001042592	1426818_at	sterol o-acyltransferase 1	Arrdc4
NM_146064	1460722_at	sterol o-acyltransferase 2	Soat2
NM_011519	1415944_at, 1448158_at	syndecan 1	Sdc1
NM_009318	1421812_at, 1450378_at	tap binding protein	Tapbp
NM_011579	1449009_at	t-cell specific gtpase	Tgtp
NM_023141	1421998_at, 1450454_at	torsin family 3, member a	Tor3a
NM_015799	1425381_a_at	transferrin receptor 2	Trfr2
NM_013683	1416016_at	transporter 1, atp-binding cassette, sub-family b (mdr/tap)	Tap1
NM_009277	1418077_at, 1448940_at	tripartite motif protein 21	Trim21
NM_009546	1426415_a_at	tripartite motif protein 25	Trim25
NM_021885	1420925_at	tubby candidate gene	Tub
NM_011610	1448951_at	tumor necrosis factor receptor superfamily, member 1b	Tnfrsf1b
NM_009396	1438855_x_at	tumor necrosis factor, alpha-induced protein 2	Tnfaip2
NM_030711	1416942_at	type 1 tumor necrosis factor receptor shedding aminopeptidase regulator	Arts1
NM_019392	1425248_a_at, 1425249_a_at	tyro3 protein tyrosine kinase 3	Tyro3
NM_021394	1419604_at, 1429947_a_at	z-dna binding protein 1	Zbp1
NM_011777	1417240_at	zyxin	Zyx
